# Enhancing Phosphorus Availability Through Bagasse Biochar Addition and Changes in *phoD* Bacterial Communities of Karst and Non-Karst Forest Soils

**DOI:** 10.3390/microorganisms14061373

**Published:** 2026-06-21

**Authors:** Yanjun Chen, Xinyu He, Yueming Liang, Fujing Pan, Cheng Zeng, Haijun Tan, Qiang Li, Zeyan Wu

**Affiliations:** 1 Guangxi Key Laboratory of Environmental Pollution Control Theory and Technology, College of Environmental and Engineering, Guilin University of Technology, Guilin 541006, China; 19816261072@163.com (Y.C.); 18303881368@163.com (X.H.);; 2 Karst Dynamics Laboratory of Ministry of Natural Resources, Institute of Karst Geology, Chinese Academy of Geological Sciences, Guilin 541004, China; yueming0919@163.com (Y.L.);; 3 University Engineering Research Center of Watershed Protection and Green Development of Guangxi, Guilin University of Technology, Guilin 541006, China; 4 Key Laboratory of Carbon Emission and Pollutant Collaborative Control, Education Department of Guangxi Zhuang Autonomous Region, Guilin University of Technology, Guilin 541006, China; 5 Guangxi Key Laboratory of Germplasm Innovation and Utilization of Specialty Commercial Crops in North Guangxi, Guangxi Academy of Specialty Crops, Guilin 541004, China

**Keywords:** karst, bagasse biochar, soil phosphorus availability, *phoD* bacteria

## Abstract

Biochar can enhance microbial-mediated organic phosphorus mineralization, but the underlying mechanisms remain unknown in forest soils with varying pH values. An incubation experiment was conducted using karst (alkaline) and non-karst (acidic) forest soils. Four amounts of bagasse biochar were applied (0, 5, 10, and 15 t·hm^−2^) to assess their effects on soil phosphorus availability and microbial community structure. Olsen-P content of alkaline and acidic forest soils increased with increasing bagasse biochar addition and incubation time, especially in non-karst forest soil. The structure and diversity of *phoD*-harboring bacterial community of acidic forest soil were significantly altered by the amount of bagasse biochar added and the incubation time, whereas those in alkaline karst forest soil were not significantly affected. The relative abundance of the dominant order Burkholderiales reached (43%) in acidic forest soil, significantly exceeding the (9%) recorded in alkaline karst forest soil. The *phoD* bacteria in acidic forest soil had more complex microbial networks and were more closely related to phosphorus fractions than those in alkaline forest soil. Structural equation modeling indicated that soil phosphorus availability was directly controlled by bagasse biochar input in acidic forest soil, with an indirect pathway linked to *phoD* bacterial community structure. The contribution of *phoD* bacteria to the variation in phosphorus availability was higher in acidic forest soil than in alkaline forest soil based on variance partitioning, indicating that enhancing soil phosphorus availability with bagasse biochar depends on the amount added, soil type, and its regulation of *phoD* bacterial communities.

## 1. Introduction

Phosphorus serves as a key macronutrient required for plant development [1]. Within soils, it occurs as organic and inorganic species. The organic phosphorus pool, which includes nucleic acids, nucleotides, sugar phosphates, inositol phosphates, and phospholipids, accounts for 30–65% of the soil total phosphorus (TP) content. Inorganic phosphorus, which comprises metaphosphates, pyrophosphates, and orthophosphates, accounts for approximately 35–70% [2]. Less than 5% of inorganic phosphorus in solution is available for direct uptake by microbes and plants [3,4]. According to meta-analytical evidence, nearly 43% of land-based ecosystems experience phosphorus scarcity that constrains plant growth [5,6]. Karst landscapes occupy roughly 15% of the global terrestrial area. In China, the southwestern karst region represents about 5.8% of the national territory and is widely regarded as one of the world’s largest continuous karst belts [7,8]. Karst ecosystems are characterized by high rock exposure, shallow and discontinuous soil layers, and soils that are alkaline and rich in calcium [9]. In soil, Olsen-P binds easily with calcium to form insoluble calcium phosphates, leading to widespread phosphorus limitation for plant growth, particularly in forest ecosystems [10].

The influence of biochar application on soil phosphorus availability has been well studied [11], and most studies have indicated that increasing biochar amount increases phosphorus availability of biochar-amended soils [1,12], especially in acidic soils [13]. This is because biochar directly provides phosphorus or desorbs insoluble organic phosphorus by altering physical and chemical soil properties [14,15]. In addition to that, biochar application increases the activity of alkaline and/or acid phosphomonoesterases in the soil [16,17]. Phosphomonoesterases promote the mineralization of organic phosphorus to inorganic phosphorus to enhance phosphorus availability [18]. Synthesis of alkaline phosphomonoesterases is regulated by *phoD* bacteria [19], which are frequently present in both acidic and alkaline soil [20]. This synthetic process is induced by phosphorus scarcity, which stimulates microorganisms to upregulate *phoD* gene expression [21]. The diversity and compositional patterns of *phoD* bacteria vary with soil pH [20,22], which may shift after biochar amendment [23]. Previous studies on acidic soils have reported that biochar-induced shifts in *phoD* bacterial diversity and assemblage composition are associated with soil phosphorus availability [24], whereas other studies in alkaline soils have shown that the diversity of *phoD* bacteria responds poorly to biochar application [25]. Although studies on biochar have expanded, how biochar interacts with *phoD* bacteria remains poorly understood. For instance, the magnitude of improvement in phosphorus availability following biochar amendment across soils with different pH conditions, as well as the role of *phoD* bacterial shifts in regulating phosphorus availability, is still uncertain. Further investigation is therefore required to clarify the links among *phoD* bacterial assemblages, phosphorus availability, and biochar-driven changes in soil pH.

Sugarcane is an important strategic resource for China. Sugarcane cultivation in Guangxi covered 835,100 ha in 2023. The sugar production reached as high as 70 million tons, accounting for 69.08% of China’s total output [26]. Approximately 28% of the sugarcane residue is produced but is not effectively utilized during the sugarcane pressing process [27,28,29]. Sugarcane bagasse biochar, produced through high-temperature pyrolysis, has been found to enhance soil structure [30,31,32,33]. Bagasse biochar added to P-limited forest soils may improve phosphorus availability and provide an effective strategy for waste utilization. This study focuses on two forest soils with distinct pH levels: alkaline karst and acidic non-karst soils. It is hypothesized that (1) addition of bagasse biochar would increase phosphorus availability in the two types (karst and non-karst), with a more significant effect in soils with greater phosphorus limitation; and (2) in alkaline karst soils, bagasse biochar would enhance phosphorus availability through physicochemical processes more than through microbial processes, compared to acidic non-karst soils, where the latter predominates.

## 2. Materials and Methods

### 2.1. Study Site

The present study was conducted in a karst forest site located in the Mulun National Nature Reserve (25°06′09″–25°12′25″ N, 107°53′29″–108°05′42″ E), whereas the non-karst forest site was located in the Huashan Forest Farm (25°06′ N, 108°15′ E), both in Huanjiang Maonan Autonomous County, Guangxi, China. Both sites are characterized by a subtropical climate, where the mean annual temperature is 16.5–20.5 °C, and yearly rainfall is approximately 1400–1500 mm. Rainfall is concentrated from April to September, and the drought period extends from October to March [34]. The dominant vegetation of Mulun Nature Reserve includes Celtis biondii, Loropetalum chinense, Cryptocarya chinensis, Pteroceltis tatarinowii, and Miliusa chunii, together with Cleidion bracteosum and Cyclobalanopsis glauca, whereas Huashan Forest Farm is mainly represented by a Pinus massoniana plantation. Other recorded species include Schefflera heptaphylla, Ficus tikoua, Vernonia solanifolia, Evodia lepta, and Rhodomyrtus tomentosa. These forests are around 30 years old and were established following the “Returning Farmland to Forest” program, which transformed former maize fields into forested areas.

Soils collected from Mulun Nature Reserve were treated as karst forest soils, whereas samples obtained at Huashan Forest Farm were regarded as non-karst forest soils. Following the international soil classification system and our previous study [35], the karst forest soils were classified as Lithosols, a Leptosol-related soil type developed on dolostone or limestone parent materials, while the non-karst forest soils were classified as Ferralsols. These two soil types occur within the same latitudinal region.

### 2.2. Soil Sample Collection

Eight sites were randomly selected from the karst and non-karst forests in the study area in August 2020, and a 20 m × 20 m plot was established at each site. The distance between any two plots exceeded 50 m. All plots were selected with consistent environmental conditions: an easterly aspect, a midslope position, and a slope of approximately 35.3°. Each 0–20 cm soil sample was obtained from ten points within a plot using an S-shaped sampling pattern. For each forest type, eight soil samples were thoroughly homogenized to generate a pooled soil sample; consequently, two pooled samples were obtained for this work. In the laboratory, the collected soils were passed through a 2 mm sieve to eliminate roots and gravel. A large part of these sieved soils was kept at 4 °C and used in the following incubation experiments within seven days. A smaller subsample was air-dried to measure basic soil physicochemical characteristics. The basic soil physicochemical properties were as follows. For the karst forest soil, pH was 7.27. Soil organic carbon (SOC), TN, and TP were 113.69, 10.49, and 1.78 g·kg^−1^, respectively. Olsen-P was 4.30 mg·kg^−1^. For the non-karst forest soil, the corresponding values were 13.14, 0.83, and 0.38 g·kg^−1^ for SOC, TN, and TP, respectively, 2.23 mg·kg^−1^ for Olsen-P.

### 2.3. Physico-Chemical Properties of Bagasse Biochar

The bagasse biochar was collected from Dongtang Xinkai Sugar Industry Co., Ltd. in Nanning, Guangxi Zhuang Autonomous Region, China. The biochar was produced through slow pyrolysis of bagasse heated from 300 °C to 600 °C with a heat increase of 8 °C min^−1^ for 2 h. The collected bagasse biochar was thoroughly mixed and separated into two subsamples. One subsample was allocated to the incubation experiments, whereas the remaining subsample was analyzed for its basic physicochemical characteristics. The basic physicochemical characteristics of bagasse biochar were as follows: specific surface area was 315.41 m^2^·g^−1^, pH was 10.21, SOC was 172.23 g·kg^−1^, TN and TP were 1.22 and 1.19 g·kg^−1^, respectively. Olsen-P was 7.83 mg·kg^−1^, and K (kalium) was 16.50 mg·kg^−1^.

### 2.4. Incubation Experiment

Four bagasse biochar addition treatments were established in the incubation experiment according to the procedure reported by Ge [36]. no addition (C0, 0 t·hm^−2^), 0.32% (C1, 5 t·hm^−2^), 0.64% (C2, 10 t·hm^−2^), and 0.96% (C3, 15 t·hm^−2^). Each treatment had four replicates. The experimental procedure was as follows: 100 g of fresh soil was homogenized with the assigned bagasse biochar dose. Each mixture was placed in a 500 mL brown incubation bottle. Soil moisture was kept at 60% of the field water-holding capacity, which was maintained using the weighing method every 3 days during the incubating period. The bottles were then kept under dark conditions at 25 °C throughout an 80-day incubation period [37]. During this period, soil samples were taken after 7, 20, 40, and 80 days to measure phosphorus fractions, enzyme activities, microbial biomass, and *phoD* bacterial community composition.

### 2.5. General Soil Parameters

The contents of pH, SOC, and microbial biomass phosphorus (MBP) were measured using the methods detailed in our previous study [38].

### 2.6. Soil Phosphorus Components

Olsen-P was obtained through the NaHCO_3_ extraction process and quantified using the molybdenum–antimony colorimetric UV spectrophotometric method. After fumigation, samples were extracted with NaHCO_3_, and color development followed the same procedure used for Olsen-P [39].

Soil phosphorus fractionation based on bioavailability was performed according to the BBP method [14]. Briefly, fresh soil (0.5 g) was placed in a 15 mL centrifuge tube, followed by the addition of 10 mL extractant. The suspension was then agitated at 180 rpm for 3 h under 25 °C. After shaking, the supernatant was collected following centrifugation. The extraction solutions used for Citrate-P and HCl-P were citrate at 10 mmol L^−1^ and hydrochloric acid at 1 mol L^−1^, respectively. For Enzyme-P, the extracting solution comprised acid phosphatase, alkaline phosphatase, and phytase, with each enzyme supplied at 0.02 EU mL^−1^. Since phytase itself contains phosphorus, it was dialyzed for 5 days at 4 °C using dialysis membranes. These four phosphorus pools were quantified with the malachite green colorimetric assay [40].

Soil phosphatase activity was quantified with the MUB fluorometric assay [41]. Fresh soil (1 g) was placed in a capped sterile glass bottle of 500 mL. Then, 125 mL sodium acetate buffer was introduced, followed by thorough shaking of the mixture. Subsequently, the sodium acetate buffer microplate was added using a pipette. The microplate was kept under dark conditions at 20 °C for 4 h. Afterward, 10 μL NaOH solution (1 mol L^−1^) was added to stop the reaction. Fluorescence intensity was subsequently recorded using a Synergy H4 microplate reader. The calculated enzyme activity is given in nmol g^−1^ h^−1^.

### 2.7. DNA Extraction and Illumina Sequencing

Genomic DNA was isolated from soil samples (0.5 g) using the FastDNA SPIN Kit (MP Biomedicals, Cleveland, OH, USA). DNA integrity and quantity were evaluated using 1% agarose gel electrophoresis and UV spectrophotometry (NanoDrop Technologies, Wilmington, NC, USA), respectively.

Target *phoD* fragments were amplified with the primer pair ALPS-F730 (5′-CAGTGGGACGACCACGAGGT-3′) and ALPS-1101 (5′-GAGGCCGATCGGCATGTCG-3′) [42]. A 25 μL PCR mixture was prepared for each sample, consisting of 2.5 μL 10× Ex Taq Buffer (Mg^2+^ plus), 0.3 μL of Ex Taq (Takara Biotechnology, Dalian Co., Ltd., Dalian, China), 1 μL of each primer (10 pM), and 1 μL DNA template, approximately 30 ng. Sterile double-distilled water was then supplied to adjust the reaction volume to 25 μL. The PCR program comprised an initial 3 min denaturation at 95 °C, followed by 95 °C for 20 s, 57 °C for 40 s, and 72 °C for 60 s, with a terminal extension at 72 °C for 5 min. The resulting PCR products were cleaned with a TIANquick Midi Purification Kit(TIANGEN, Beijing, China). The constructed amplicon libraries were subsequently sequenced on an Illumina NovaSeq 6000 system by Magigene Co., Ltd. (Guangzhou, China). Raw sequence reads were processed in QIIME2 [43], and reads shorter than 200 bp or containing ambiguous bases were filtered out. Chimeric reads were further excluded with USEARCH in the QIIME2 workflow. Reads that failed to correspond to *phoD* or contained stop codons were removed with FrameBot in the RDP functional gene pipeline. The retained high-quality reads were grouped into operational taxonomic units (OTUs) at 97% sequence similarity with UCLUST. Taxonomic identities of these OTUs were assigned by BLAST searches against the Greengenes database [44].

### 2.8. Statistical Analysis

Before statistical analysis, all datasets were examined for normality and variance homogeneity using SPSS 27.0. One-way ANOVA combined with Tukey’s Honestly Significant Difference (HSD) test was applied to compare basic soil physicochemical properties, phosphorus fractions, and phosphatase activities under different bagasse biochar addition amounts and incubation times. The R (version 4.4.2) vegan package was applied for the aforementioned correlations among soil phosphorus variables and, separately, to assess the effects of various soil factors on Olsen-P dynamics following biochar addition in the contrasting forest soils. Random forest models were constructed with the random forest and rfPermute packages to identify the key factors influencing soil Olsen-P content in karst and non-karst areas.

Before network construction, OTUs showing less than 0.1% relative abundance were excluded [45]. After Benjamini–Hochberg (BH) correction, Spearman correlations were calculated, and edges were retained when r > 0.6 and phosphorus < 0.05. Network nodes and edges were defined using the psych package, and the networks were visualized in Gephi 0.10. A correlation network between *phoD* taxa and soil phosphorus fractions was then built using the psych package. Core taxa were selected using the following criteria: degree above 50, closeness centrality above 0.44, and betweenness centrality below 0.12. Network diagrams were then constructed based on these criteria. Orthogonal Partial Least Squares Discriminant Analysis (OPLS-DA) was conducted with SIMCA 14.1 based on *phoD* OTU profiles. This analysis served to display shifts in the *phoD* bacterial assemblage under varying bagasse biochar doses and incubation durations across karst and non-karst soils.

Structural equation modeling (SEM) was applied to assess causal links among bagasse biochar addition amount, pH, phosphatase activity, *phoD* bacteria, bioavailable phosphorus fractions, and Olsen-P. Model adequacy was assessed using the χ^2^ statistic, df, GFI, AGFI, and RMSEA, representing the goodness-of-fit index, adjusted goodness-of-fit index, and root mean square error of approximation, respectively.

## 3. Results

### 3.1. Effects of Bagasse Biochar Addition on Soil pH, Phosphorus Fractions, and Enzyme Activities

**Figure 1:**
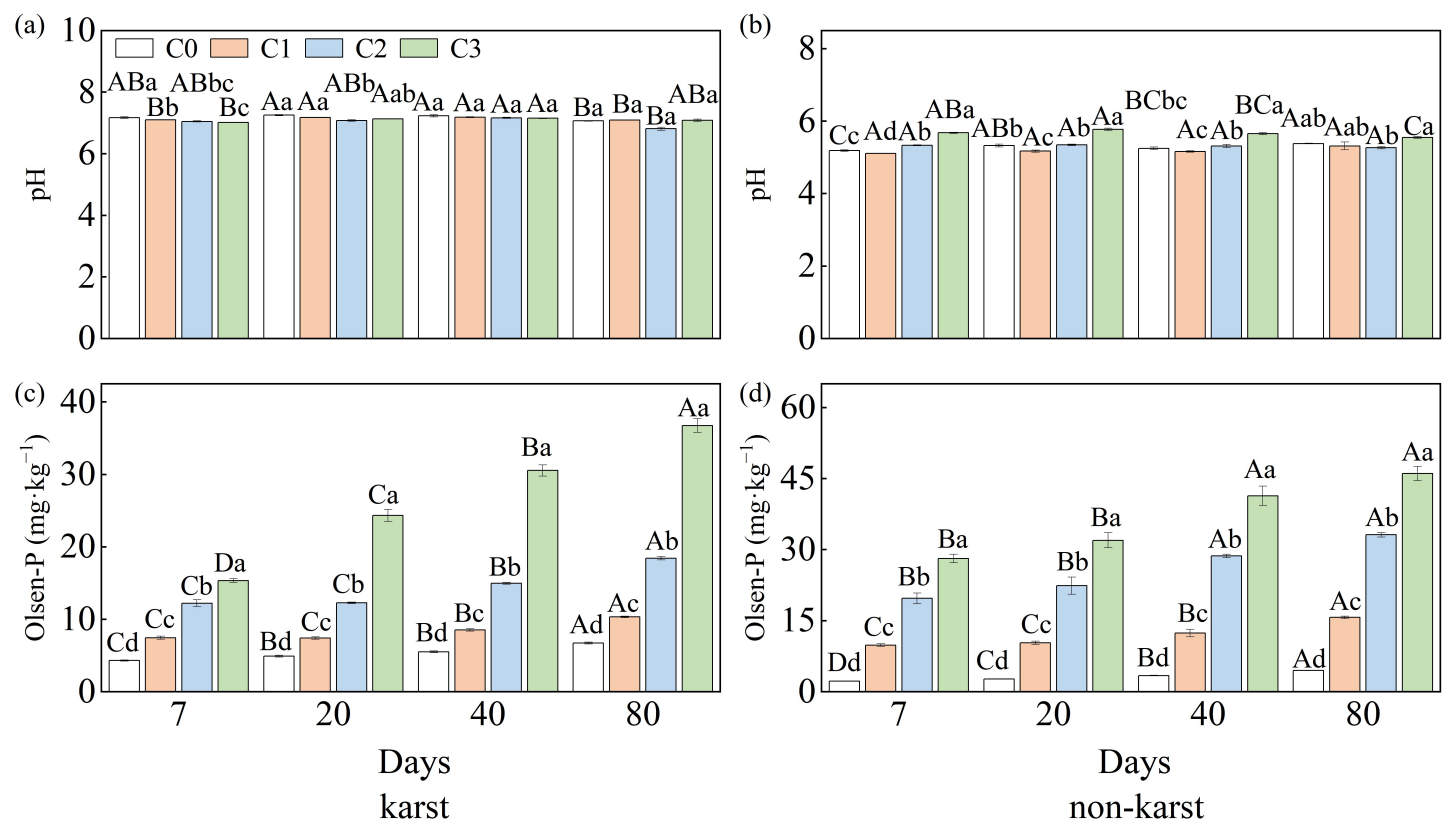
Soil pH (**a**,**b**), Olsen-P (**c**,**d**) content measured of two soil types and amended with biochar at application amounts of 0, 5, 10, and 15 t·hm^−2^ (C0, C1, C2, C3, respectively). Different capital and lower letters indicate significant differences among different culture days at four biochar treatments.

**Figure 2:**
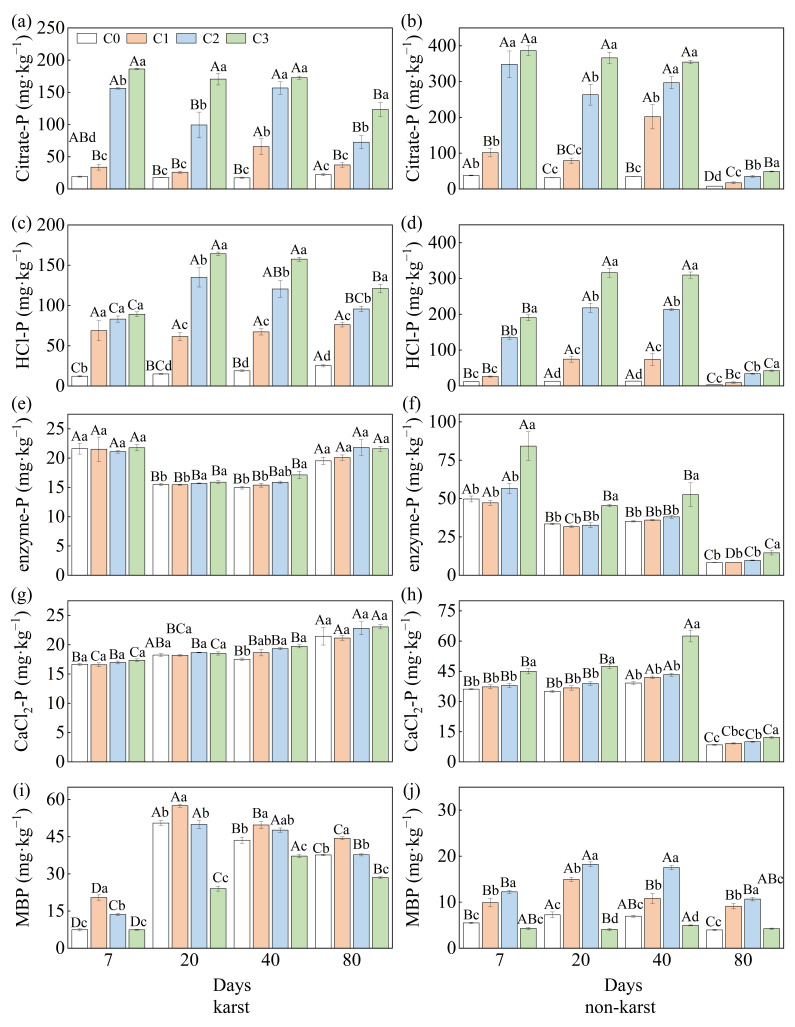
Soil Citrate-P (**a**,**b**), HCl-P (**c**,**d**), enzyme-P (**e**,**f**), CaCl_2_ (**g**,**h**), and MBP (**i**,**j**) content measured in two soil types and amended with biochar at application amounts of 0, 5, 10, and 15 t·hm^−2^ (C0, C1, C2, C3, respectively). Different capital and lower letters indicate significant differences among different culture days at four biochar treatments.

At the same incubation time, the pH of karst forest soil was markedly lowered via bagasse biochar addition in comparison with that in the control, except on day 80. In non-karst forest soil, pH initially fell and subsequently climbed as the amount of added bagasse biochar increased. For the same amount of bagasse biochar, the pH in karst forest soil first rose, subsequently fell with increasing incubation time. For non-karst forest soil, only treatment C3 displayed this pattern. (Figure 1a,b). For the same incubation time, Olsen-P rose with increasing bagasse biochar addition across the two soil types. With the same amount of bagasse biochar added, Olsen-P rose with incubation time in both soils (Figure 1c,d). For the same incubation time, citrate-P and HCl-P were strongly enhanced by bagasse biochar across the two soil types (karst and non-karst). For the same amount of bagasse biochar, citrate-P peaked on day 7 and reached its minimum on day 80 in both soils. The HCl-P reached its maximum level on day 20 (Figure 2a–d). For the same incubation time, enzyme-P significantly rose in the C3 treatment in comparison with the control across the two soils, except for day 7 of the karst soil. With the same amount of bagasse biochar added, enzyme-P peaked on day 7 in both soils. The minimum enzyme-P concentration occurred on day 20 in karst forest soil, and on day 80 in non-karst forest soil (Figure 2e,f). For the same incubation time, CaCl_2_-P showed no significant change with the bagasse biochar amount added into the karst forest soil. CaCl_2_-P significantly rose with the addition of amounts of bagasse biochar (C3) of non-karst forest soil. CaCl_2_-P rose with increasing incubation time with the same amount of bagasse biochar added, except in karst soil on day 80 (Figure 2g,h). At the same incubation time, MBP for C1 and C2 treatments significantly rose in comparison with the control in both soils, whereas MBP for C3 significantly fell. For the same bagasse biochar addition amount, the MBP initially rose and subsequently fell over time in the two soil types, reaching its highest on day 20 (except for in the C3 treatment; Figure 2i,j).

For the same incubation time, ACP activity of karst forest soil showed no significant change with bagasse biochar amount added, compared to the control (Table 1). ACP activity of non-karst forest soil significantly fell with the amount of added bagasse biochar increased, except on day 7. For the same addition of bagasse biochar, ACP activity decreased with increasing incubation time in karst forest soil. The pattern of ACP activity of non-karst forest soil was not obvious.

For the same incubation time, the ALP activity of karst forest soil significantly rose under C1 and C2 treatments on day 7 but was significantly decreased on day 20 (Table 1). The ALP activity showed no significant change with bagasse biochar addition on day 40 or 80. In non-karst forest soil, ALP activity was not significantly influenced by the bagasse biochar amount added. For the same bagasse biochar addition amount, ALP activity in the karst soil initially fell and subsequently climbed over time, with the highest value on day 20. The pattern of ALP activity in the non-karst soil showed an “N” shape, with the highest value on day 80.

### 3.2. Community Structure and Diversity of phoD Bacteria

**Figure 3:**
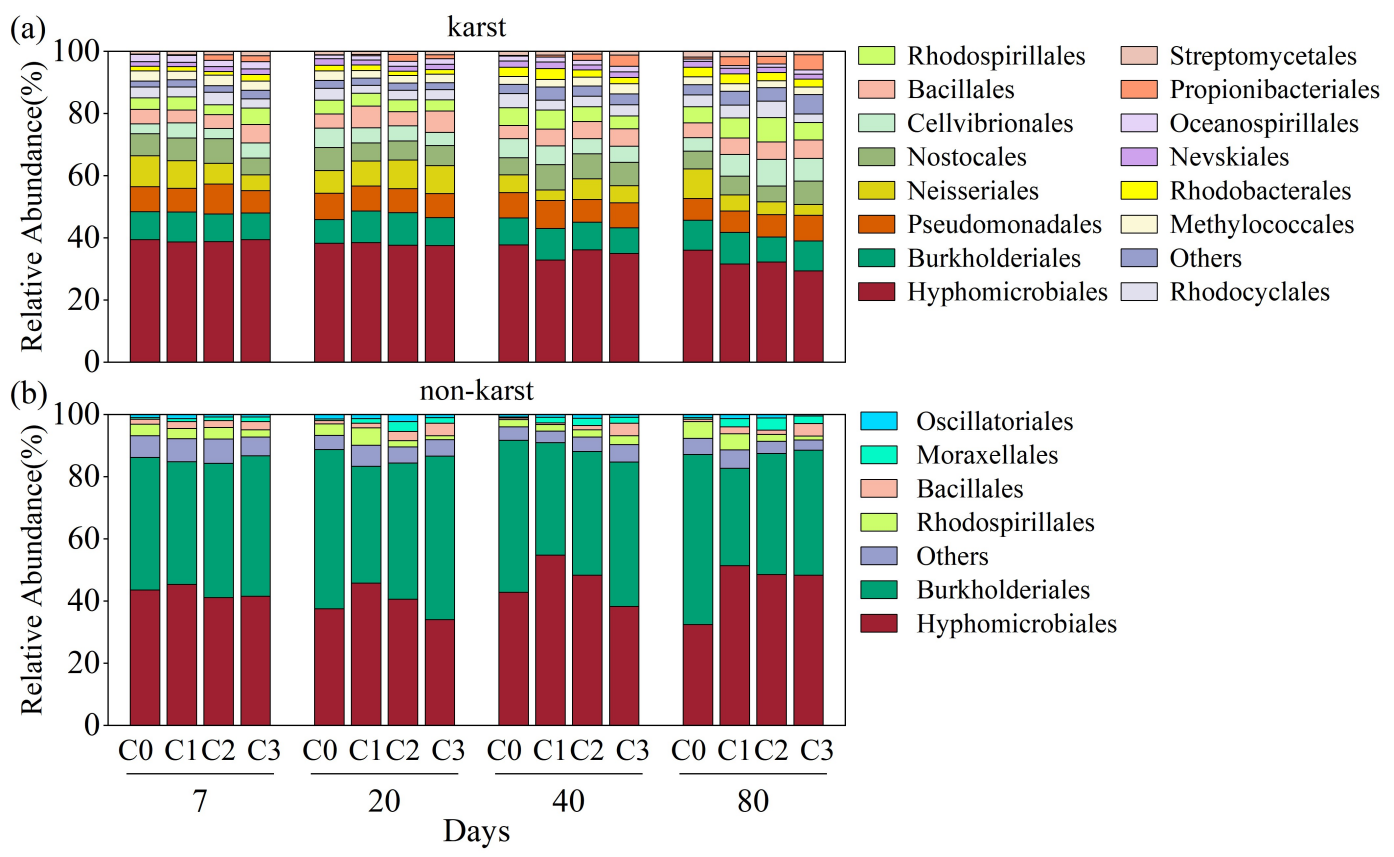
Taxonomic composition of *phoD* bacteria communities at the order level of two soil types, karst (**a**) and non-karst (**b**), with bagasse biochar amended at application amounts of 0, 5, 10, and 15 t·hm^−2^ (C0, C1, C2, C3, respectively).

In karst forest soils, Hyphomicrobiales (36%), Burkholderiales (9%), and Pseudomonadales (7.9%) were the dominant orders. Together, they accounted for approximately 50%. The relative abundance of Propionibacteriales increased with both longer incubation times and higher amounts of bagasse biochar added. No significant differences were detected for the other two orders across treatments. For the same incubation time, the relative abundance of Streptomycetales, which was not a dominant order, decreased as the amount of bagasse biochar increased, except on day 7. For the same amount of bagasse biochar added, the relative abundance of Streptomycetales increased with incubation time (Figure 3a).

In non-karst forest soil, Hyphomicrobiales (43%) and Burkholderiales (43%) were the dominant orders. Together, these accounted for approximately 80% of communities at the order level. For the same incubation time, the relative abundance of Hyphomicrobiales initially increased, subsequently decreased as the amount of bagasse biochar added increased, reaching its highest at the C1 treatment. Compared to the control, the relative abundance of Hyphomicrobiales under C1 treatment was 4%, 22%, 28%, and 58% higher at different incubation times. However, it was only weakly affected by the incubation time. For the same incubation time, the relative abundance of Burkholderiales initially decreased, subsequently increased as the amount of bagasse biochar added increased, with the lowest value observed in the C1 treatment. On days 7 and 20, the amount of bagasse biochar added did not significantly affect relative abundance. On days 40 and 80, biochar addition reduced the relative abundance. With the same amount of bagasse biochar added, Burkholderiales were not significantly affected by the incubation time (Figure 3b).

Although Hyphomicrobiales and Burkholderiales were the dominant orders in the two soil types, their combined relative abundance was higher in non-karst soils (86%) than in the other (45%). Furthermore, these microbial groups exhibited greater sensitivity to bagasse biochar levels in non-karst soils. The *phoD* bacterial diversity indices in karst forest soil showed no significant change with bagasse biochar amount added or incubation time. However, a significant effect was observed in non-karst forest soil. With special focus on non-karst forest soil, at the same incubation time, the OTUs and Chao1 indices increased with the amount of added bagasse biochar in the C1 and C2 treatments. Both the Shannon and Simpson indices also increased with the amount of added bagasse biochar, although the improvement was similar across the different application amounts. With the same amount of bagasse biochar added, the OTUs and Chao1 indices decreased with increasing incubation time. The Shannon index decreased only under the C3 treatment with longer incubation periods, whereas the Simpson index showed no significant changes over time (Figure S1).

**Figure 4:**
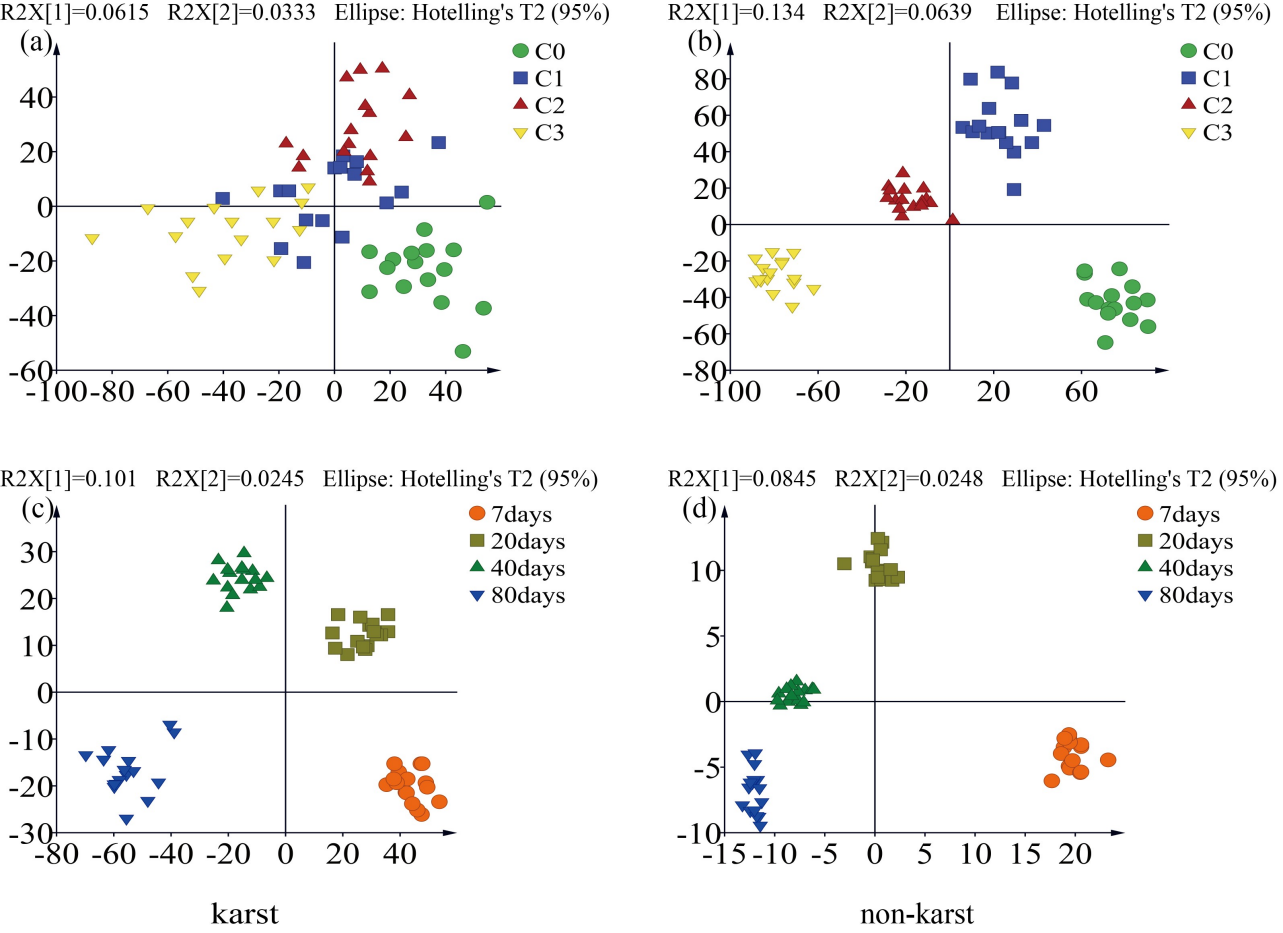
OPLS-DA analysis of *phoD* bacteria communities at OTU lever of two soil types, karst (**a**) and non-karst (**b**), with biochar amended at application amounts of 0, 5, 10 and 15 t·hm^−2^ (C0, C1, C2, C3, respectively); Karst soil (**c**) and non-Karst soils (**d**) with different incubation time of 7, 20, 40, 80 days.

**Figure 5:**
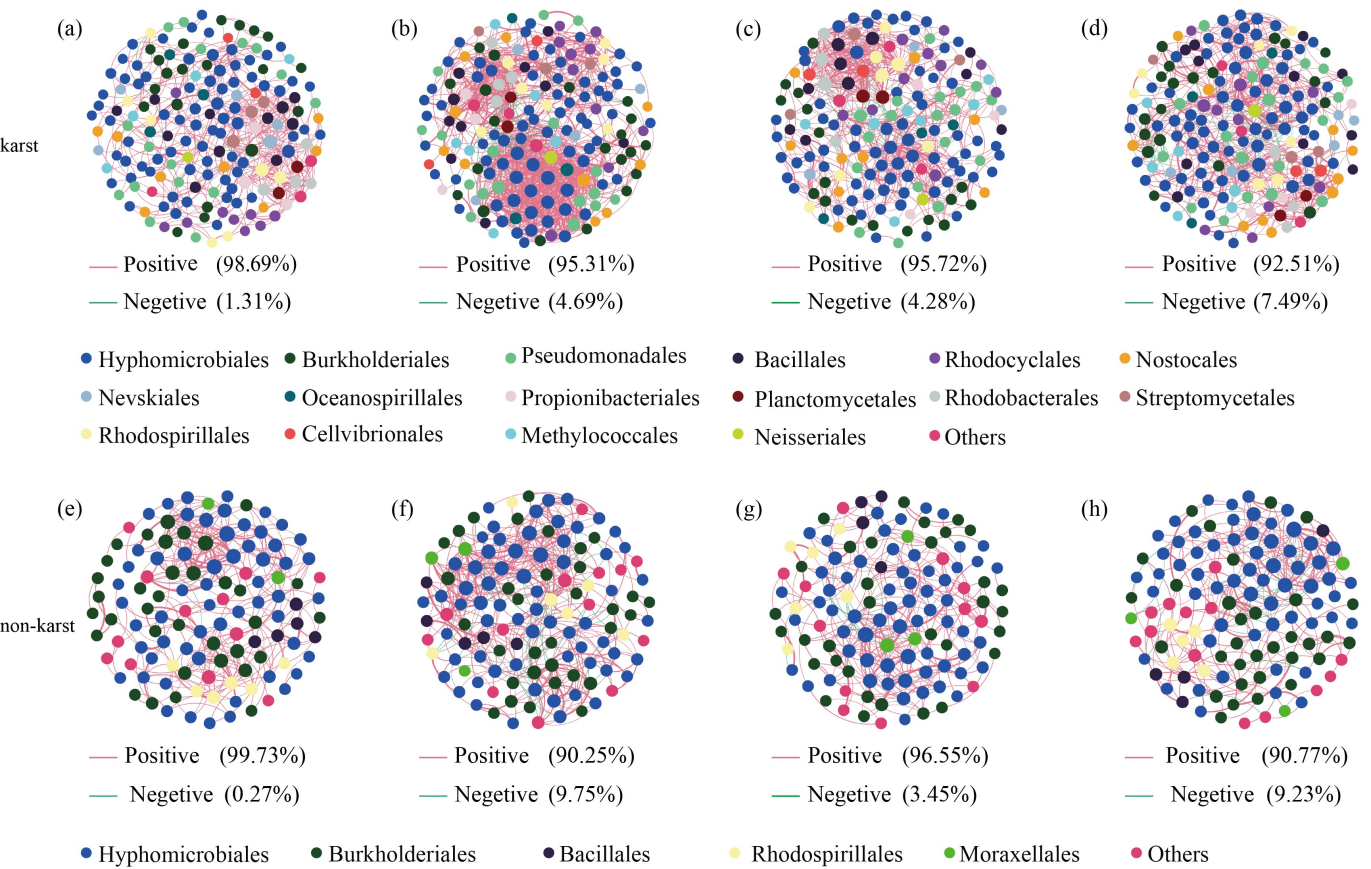
Co-occurrence networks of *phoD* bacteria of two soil types, karst (**a**–**d**) and non-karst (**e**–**h**), under bagasse biochar addition at amounts of 0, 5, 10, and 15 t·hm^−2^. Red lines represent positive interactions, and green lines represent negative interactions.

Based on OPLS-DA profiling, the community of *phoD* bacteria in the two soil types was significantly correlated with bagasse biochar amount addition and the incubation time, with the temporal factor showing a stronger correlation than the amount of bagasse biochar added in the karst (Figure 4). The microbial network co-occurrence graph showed that at the same incubation time, for the two soil types, node number, edge number, and the graph density initially increased, subsequently decreased with increasing bagasse biochar addition. The number of positively correlated edges decreased as the number of additions increased. In contrast, module number, average clustering coefficient, and average path length exhibited opposing trends with increasing addition amounts (Figure 5; Table S1). For the same amount of bagasse biochar addition, node number, edge number, graph density, and average clustering coefficient initially increased, subsequently decreased with increasing incubation time. Average path length initially decreased, subsequently increased with increasing incubation time. Positively correlated edge number and module number decreased with increasing incubation time, except on day 80. For non-karst forest soil, node number, edge number, average degree, graph density, and average clustering coefficient increased with increasing incubation time. The number of positively correlated edges and modules decreased with increasing incubation time (Figure S2; Table S2). For karst forest soil, node number, module number, the number of positively correlated edges, network diameter, average clustering coefficient, and average path length were higher than those for non-karst forest soil. However, edge number and graph density were lower than those of the non-karst soil (Figure S3; Table S3).

### 3.3. The Relationship Between phoD Bacteria and Soil Phosphorus Fractions

**Figure 6:**
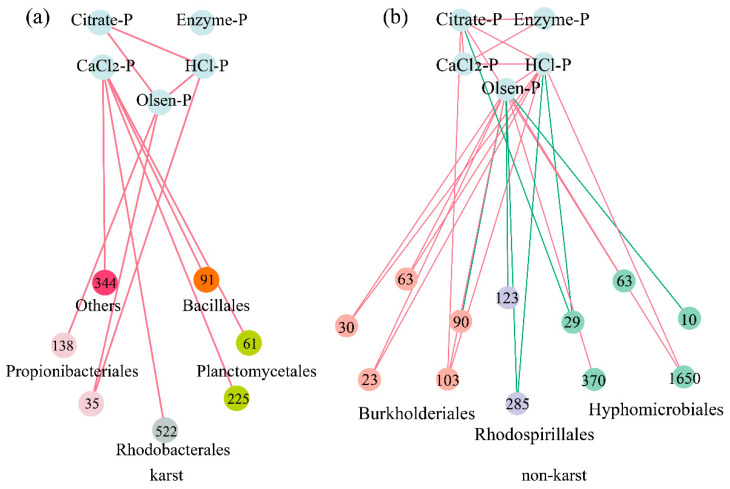
Correlation networks of core functional groups of *phoD* bacteria with phosphatases and phosphorus fractions of two soil types, karst (**a**) and non-karst (**b**). Red lines represent positive interactions, and green lines represent negative interactions. Core functional groups were identified according to the following criteria: (1) relative abundance of OTUs > 0.1%; (2) OTUs with a degree > 50; (3) betweenness centrality < 0.12; (4) closeness centrality > 0.44.

The co-occurrence networks of OTUs, phosphorus fractions, and Olsen-P showed that nodes were selected using the criteria of betweenness centrality < 0.12, closeness centrality > 0.44, and relative OTU abundance > 0.1%. Seven OTUs were identified as core functional bacteria of karst forest soil, mainly belonging to Bacillales, Planctomycetales, Rhodobacterales, Propionibacteriales, and one unclassified order. Twelve OTUs were identified as core functional bacteria of non-karst forest soil, mainly belonging to Burkholderiales, Rhodospirillales, and Hyphomicrobiales. Positive correlations were observed between the core bacterial groups and phosphorus fractions in the two soil types (karst and non-karst). Positive correlations were mainly associated with Burkholderiales and Hyphomicrobiales, whereas negative correlations were found between Rhodobacterales and phosphorus fractions (Figure 6).

**Figure 7:**
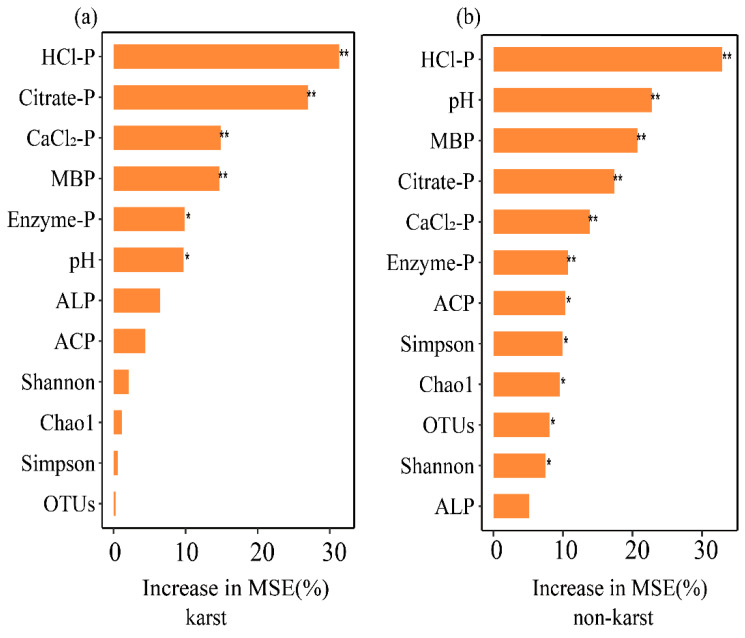
Environmental and microbial factors influencing phosphorus availability in the two soil types, karst (**a**) and non-karst (**b**). Note: ** *p* < 0.01; * *p* < 0.05.

The random forest model indicated that in the karst soil, Olsen-P content was significantly affected by HCl-P, citrate-P, CaCl_2_-P, MBP, enzyme-P, and pH (Figure 7a). In non-karst soil, the Olsen-P content was significantly affected by HCl-P, pH, MBP, citrate-P, CaCl_2_-P, enzyme-P, ACP, Simpson, Chao1, OTUs, and Shannon indices (Figure 7b).

**Figure 8:**
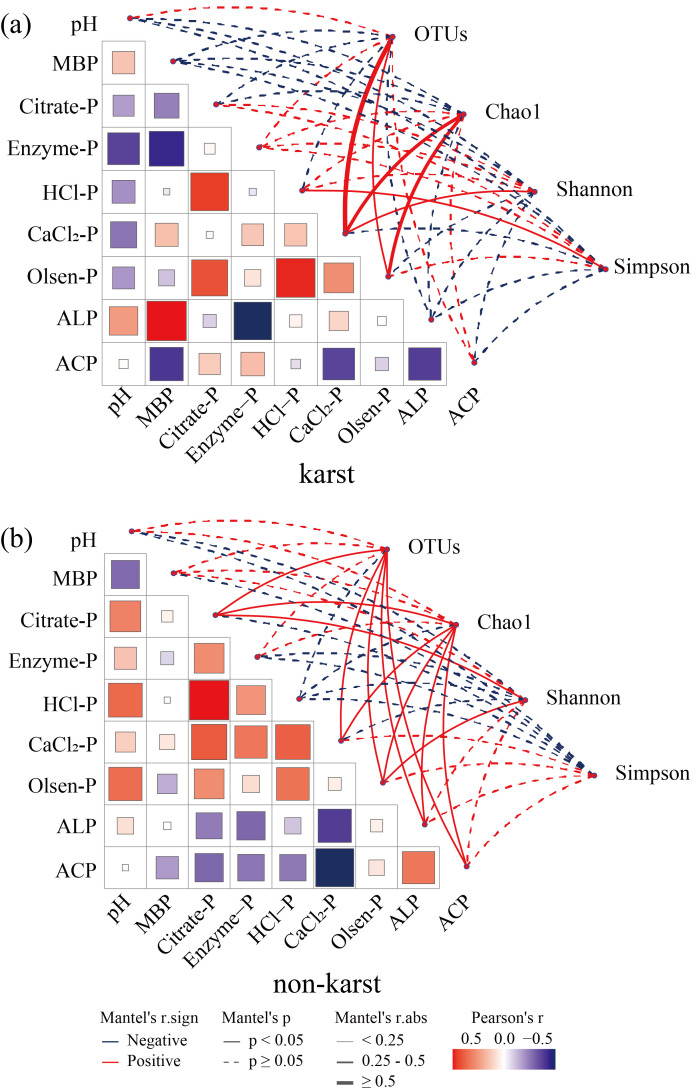
Relationships between soil physicochemical properties, phosphatase activity, and the diversity of *phoD* bacteria of two soil types, karst (**a**) and non-karst (**b**).

The Mantel test indicated that, in karst forest soil, the Simpson index exhibited significant positive correlation with HCl-P, OTUs, and the Chao1 index exhibited significant positive correlation with Olsen-P and CaCl_2_-P, respectively, and the Shannon index was significantly positively correlated with CaCl_2_-P (Figure 8). In non-karst forest soil, OTU, Chao1, and Shannon indices of the *phoD* bacterial community exhibited significant positive correlation with citrate-P and Olsen-P. OTU and Chao1 indices exhibited a significant positive correlation with CaCl_2_-P (Figure 8).

**Figure 9:**
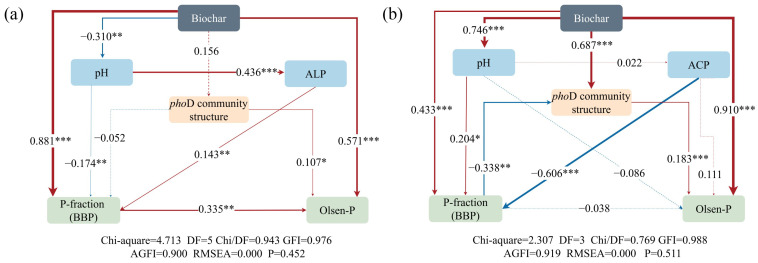
SEM showed the relationships among bagasse biochar addition, *phoD* bacteria, *phoD* bacteria community structure, Olsen-P, phosphorus fractions, and environmental variables for the two soil types, karst (**a**) and non-karst (**b**). The red arrow indicates a positive path coefficient; the blue indicates a negative path coefficient. Significance levels are indicated as follows: *** *p* < 0.001; ** *p* < 0.01; * *p* < 0.05.

The SEM showed that the addition of bagasse biochar, *phoD* bacteria, and phosphorus fractions directly affected Olsen-P in the two soil types, and a positive correlation was found between these direct effects (a negative correlation was observed between phosphorus fractions and Olsen-P of non-karst forest soil). In addition, the amount of bagasse biochar added indirectly affected Olsen-P through the pH, ACP/ALP, and phosphorus fractions. The relationship between the amount of added bagasse biochar and the phosphorus fractions was negative in karst forest soil, but these fractions were positive in non-karst forest soil (Figure 9).

**Figure 10:**
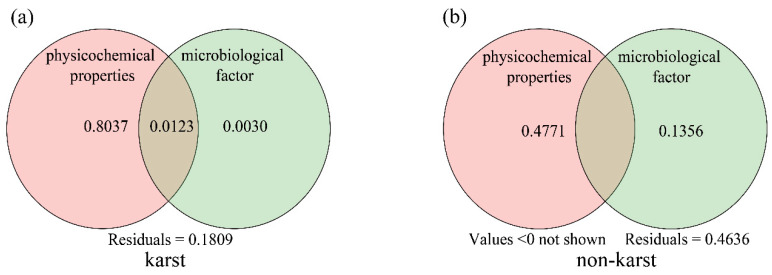
Variance partitioning analysis shows the relative contributions of physicochemical properties and microbial factors to soil Olsen-P in the two soil types, karst (**a**) and non-karst (**b**).

Variance partitioning analysis revealed that in karst forest soil, soil physicochemical properties and microbial factors independently explained 0.8037 and 0.0030 of the variation in soil phosphorus availability, respectively. The interaction term was 0.0123. Unexplained variation accounted for 0.1809. In non-karst forest soil, physicochemical properties and microbial factors independently explained 0.4771 and 0.1365 of the variation, respectively. The unexplained variation was 0.4636 (Figure 10).

## 4. Discussion

### 4.1. Effects of Bagasse Biochar Addition on Soil pH, Phosphorus Fractions, and Enzyme Activities

The pH of karst alkaline forest soil was negligibly influenced by the quantity of bagasse biochar that had been added, but was significantly increased in non-karst acidic forest soil, especially at higher soil pH levels. This finding is supported by those of Yuan [46]. This indicates that soil pH following bagasse biochar addition strongly depended on the properties and amount of bagasse biochar added, and on the initial soil pH [13]. Bagasse biochar is rich in alkaline cations (Ca^2+^, Mg^2+^, K^+^, Na^+^, etc.), which are able to exchange H^+^ and Al^3+^ that are adsorbed onto soil particles, thus raising soil pH [47,48,49]. Additionally, bagasse biochar is furnished with negatively charged functional groups (phenolic, carboxylic, and hydroxyl) that are able to chelate H^+^, increasing soil pH [50]. Such an effect is especially prominent in acidic soils [13].

Bagasse biochar affects soil Olsen-P through ligand exchange, competitive sorption, and direct phosphorus release. Biochar performs ligand exchange by using its surface functional groups (e.g., –OH, –COOH) to replace hydroxyl or other ligands from phosphate complexes, thereby forming inner-sphere complexes that alter phosphorus solubility [51]. Biochar also engages in competitive sorption, in which its abundant surface sites and charges outcompete soil minerals for phosphate binding, thus reducing phosphorus fixation and increasing Olsen-P concentration in solution [52]. Furthermore, biochar is a slow-release nutrient source and directly releases phosphorus nutrients [12]. Therefore, adding bagasse biochar significantly increases soil available phosphorus.

The Olsen-P content rose significantly within the two soil types following bagasse biochar addition. Moreover, Olsen-P content rose with the incubation period as well as the dosage of bagasse biochar added, which is in agreement with the findings of Mao [53]. Furthermore, a significantly higher increase in Olsen-P content was observed in non-karst acidic forest soil than in karst alkaline forest soil, with the former showing a 680% increase and the latter a 200% rise (Table S4). The main reasons are as follows. First, the bagasse biochar added is a direct source of soluble phosphorus, and as the application amount and incubation time increase, the phosphorus input to the soil also increases [12]. Second, the amount of bagasse biochar added indirectly affects the Olsen-P content by altering the soil pH [54]. It has been shown by prior research that at pH 6.5, phosphorus availability is highest [55]. After adding bagasse biochar, the pH of non-karst acidic forest soil increased from around 5.0 to 5.7, while in karst alkaline forest soil, pH decreased from around 7.2 to 7.0. This is why the Olsen-P content was increased more considerably in non-karst acidic soils than in the other soils upon the addition of bagasse biochar. Third, insoluble inorganic phosphorus was activated through acidification following the addition of bagasse biochar [47]. Our results showed that Olsen-P of karst alkaline forest soil was strongly associated with citrate-P, whereas in non-karst acidic forest soil, a significant correlation with HCl-P was observed, indicating that insoluble inorganic phosphorus can be activated into soluble forms through acidification following bagasse biochar addition. Fourth, phosphatase activity is affected by the amount of bagasse biochar added, which regulates the mineralization of organic phosphorus into inorganic phosphorus [56]. Therefore, it improves soil phosphorus availability. Generally, lower soil Olsen-P content causes stimulation of phosphatase secretion and promotion of the mineralization of organic phosphorus to inorganic phosphorus. Conversely, higher soil Olsen-P content inhibits phosphatase secretion [57]. At the beginning of the incubation period, with a low soil Olsen-P, the activities of ACP and ALP were both increased by the amount of bagasse biochar added (Table 1). In the later stages under high soil Olsen-P content, the ACP activity in acidic soils and the ALP activity in alkaline soils were both suppressed by the addition of bagasse biochar (Table 1).

### 4.2. The Impact of Bagasse Biochar Addition on the Structure and Diversity of phoD Bacteria

Hyphomicrobiales and Burkholderiales were both documented as the dominant orders in the two soil types. These microbial groups are typically found in soils with low Olsen-P and can serve as biomarkers of soil phosphorus availability [58]. In non-karst acidic soils, their relative abundances were markedly higher than those of karst alkaline soils. This indicates that phosphorus limitation was more severe in non-karst forest soil, a conclusion supported by the finding that the Olsen-P level measured in that soil was considerably lower than in karst forest soil (Figure 1c,d). Notably, in non-karst acidic soil, the relative abundance of Burkholderiales was roughly five times higher than in karst alkaline soil. (Figure 3). This suggests that Burkholderiales matter in enhancing phosphorus availability in acidic soils, which accords with the research of Dludlu [59]. Furthermore, it has been shown by prior research that Burkholderiales possess nitrogen-fixing potential [60], playing an important role in alleviating nitrogen limitation in non-karst acidic forest soils.

The *phoD* bacterial community structure was markedly altered by adding varying amounts of bagasse biochar. (Figure 4a,b). With more bagasse biochar added, the relative abundances of Propionibacteriales and Rhodobacterales initially increased and then decreased. This may be because nutrients were supplied to the soil through bagasse biochar addition, promoting microbial growth. However, when soil nutrient levels reach the threshold required for microbial growth, further increases in the amount of bagasse biochar may inhibit microbial growth. At the same time, these microorganisms showed close relationships in the network. The microbial network reflects the interactions between microorganisms. A higher number of positive correlations indicated stronger synergistic interactions, whereas more negative correlations suggested stronger antagonistic interactions between microorganisms [61]. This study found that the complexity of microbial networks was greater in non-karst forest soil than in karst forest soil (Figure S3; Table S3). This is likely because the Olsen-P content is lower in non-karst soil than in the other, and microorganisms strengthen the cooperation between populations to resist phosphorus stress. Similarly, Olsen-P content was enhanced by the amount of bagasse biochar added, compared to the control group, leading to reduced synergistic interactions between *phoD* bacteria in the two soil types (karst and non-karst). Furthermore, with increasing amounts of bagasse biochar, the synergistic effect initially strengthened and then weakened. This suggests that interspecific cooperation is promoted under conditions of low soil phosphorus availability. As nutrient levels increase, dominant species take over, suppressing the number of non-dominant species and intensifying competition.

This study found that the *phoD* bacterial community structure was markedly altered by incubation time (Figure 4c,d), which is in agreement with the research of Yuan [46]. On day 7 of incubation, the *phoD* bacterial community structure differed significantly from that for other incubation times, and the microbial network was simpler (Figure S2; Table S2). This is likely because the soil disturbance was higher at the start of the incubation experiment, leading to intense competition among *phoD* bacteria for survival opportunities. With increasing incubation time, the environment became more stable, and *phoD* bacteria shifted towards synergistic interactions, performing stable ecological functions. Additionally, with longer incubation time, the relative abundances of Propionibacteriales and Rhodobacterales of karst forest soil and of Hyphomicrobiales in non-karst forest soil were observed to increase. The reason for this may be that these three *phoD* bacterial groups are copiotrophic microorganisms; bagasse biochar rich in substrates for microbial growth likely accelerated the reproduction of these microorganisms as the incubation time extended.

The structure of the *phoD* bacterial community in soil is indirectly modulated by bagasse biochar via alterations in soil physicochemical properties [62], particularly pH [63]. Soil pH can directly influence the community composition of *phoD* bacteria or indirectly affect it by influencing soil nutrients [64]. Previous studies have shown that at pH levels of6–10, Hyphomicrobiales are dominant in soils, whereas at pH levels around 5–6, Burkholderiales are dominant [65,66]. This study found that although the pH of karst alkaline forest soils (approximately pH 7) and non-karst acidic forest soils (approximately pH 6) differed by approximately 1 unit, the relative abundance of Burkholderiales showed significant differences, with non-karst acidic forest soils showing significantly higher levels than karst alkaline soils. This suggests that the background pH has a significant impact on Burkholderiales. Furthermore, microbial habitat is affected by the amount of bagasse biochar added through alterations in the soil physicochemical properties, thereby exerting either positive or negative effects on microorganisms. In addition, compared with the karst forest soil, greater associations with the bioavailable phosphorus fractions and Olsen-P were exhibited by the species that dominated the non-karst forest soil (Figure 6). This suggests that the dominant species plays a more significant role in non-karst soils, where phosphorus limitation is stronger.

### 4.3. Effects of Bagasse Biochar Addition on Soil Phosphorus Availability and Implications for Future Management

**Figure 11:**
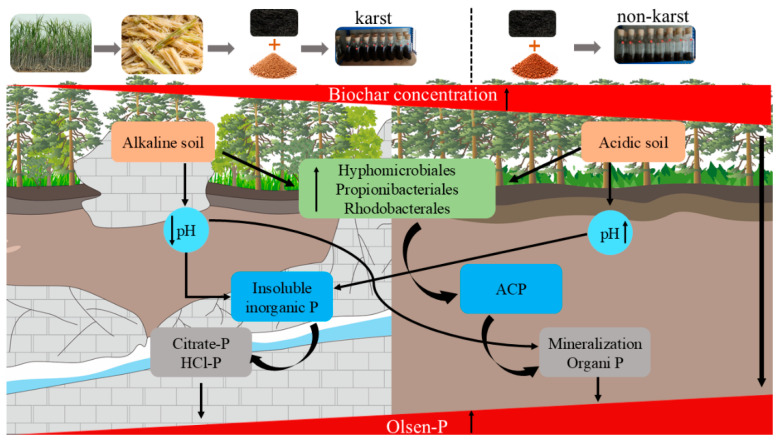
Bagasse biochar application enhances phosphorus availability in both alkaline and acidic soils.

According to a meta-analysis, 70% of global ecosystems are limited by low soil phosphorus availability [67]. Biochar is commonly applied as a soil conditioner in both natural and agricultural ecosystems. It considerably improves the Olsen-P content in soils [12,68]. Two soil types were selected for this study. These soils were severely limited by P, and they differed greatly in properties, particularly soil pH. These soils were selected to investigate the mobilization of Olsen-P following the addition of bagasse biochar. We hypothesized that the mechanism by which bagasse biochar activates phosphorus is as follows: (1) changing soil pH directly or indirectly would affect the dissolution of insoluble inorganic phosphorus into soluble inorganic phosphorus, and (2) altering the *phoD* bacterial community structure would influence the mineralization of organic phosphorus to inorganic phosphorus (Figure 11).

This study found that soil pH, Olsen-P, citrate-P, and HCl-P increased rapidly after the addition of bagasse biochar, which is rich in alkaline metal ions, to non-karst acidic forest soils. This suggests that the increase in soil pH arising as a result of bagasse biochar addition facilitates the conversion of insoluble inorganic phosphorus into soluble forms in acidic soils. At the initial stage of incubation, *phoD* bacteria survived in an environment with a low Olsen-P content. However, with the addition of bagasse biochar, they gained energy, which promoted their proliferation. The relative abundances of Propionibacteriales and Rhodobacterales in alkaline soil and Hyphomicrobiales in acidic soil increased progressively with increasing amounts of bagasse biochar added, thereby likely facilitating phosphatase secretion, which has the ability to mineralize organic phosphorus into inorganic forms. Variance partitioning analysis further quantified the relative contributions of the physicochemical and microbial processes to the activation of Olsen-P after the addition of bagasse biochar. Physicochemical processes contributed more to soil phosphorus activation than microbial processes in both acidic and alkaline forest soils. However, the effect of *phoD* bacteria on Olsen-P was greater in acidic forest soil than in alkaline forest soil. Structural equation modeling further showed that, compared with alkaline forest soil, the amount of bagasse biochar added not only directly increased phosphorus availability in acidic forest soil, but also indirectly affected phosphorus availability through *phoD* bacteria. The *phoD* gene is considered a P-starvation gene and therefore plays a stronger role when soil Olsen-P is low, whereas its expression is suppressed when soil Olsen-P is high [22].

The present incubation experiment could not fully reproduce the complexity of the natural field. This study mainly focused on the overall *phoD* bacterial community, whereas specific microbial taxa involved in phosphorus transformation were not investigated in detail. Future research should therefore include long-term field trials. Integrating plant phosphorus uptake with analyses of multiple P-cycling functional genes and specific microbial groups will help to further clarify the persistence and mechanisms of biochar-induced improvements in soil phosphorus availability.

## 5. Conclusions

The bagasse biochar addition increased soil phosphorus availability, with a stronger correlation in acidic forest soils than in alkaline forest soils. Meanwhile, the bagasse biochar addition varied in the *phoD* bacterial communities, such as changes in community structure, diversity, and network complexity. The contribution of *phoD* bacteria to the variation in phosphorus availability was likewise greater in acidic forest soil. These results indicate that the effect of bagasse biochar on improving soil phosphorus availability is strongly dependent on the soil type. Initial soil properties, especially pH, not only directly affect phosphorus speciation, but also determine the strength of the bagasse biochar effect by regulating microbial response patterns, thereby deepening our understanding of soil–microbe–nutrient interactions. The potential of bagasse biochar to improve soil phosphorus availability was also highlighted, although long-term field experiments are still needed to verify the persistence and stability of its effects and to evaluate its overall benefits in complex natural ecosystems. However, the effects of biochar on phosphorus availability through ligand exchange, competitive sorption, and direct phosphorus release were not examined in this study, and these aspects merit further investigation.

## Figures and Tables

**Table 1:** Potential ACP and ALP of two soil types are cited.

Treatment		ACP (nmol g^−1^h^−1^)				ALP (nmol g^−1^h^−1^)			
		Day 7	Day 20	Day 40	Day 80	Day 7	Day 20	Day 40	Day 80
Karst soil	C0	482.16 ± 23.98 Aa	352.75 ± 33.63 Ba	397.69 ± 10.72 ABa	310.20 ± 3.19 Ba	268.99 ± 8.04 Cab	677.05 ± 39.11 Aa	568.66 ± 14.09 Ba	480.99 ± 20.08 Ba
	C1	501.54 ± 18.55 Aa	311.15 ± 11.00 Ca	429.61 ± 9.35 Ba	324.07 ± 23.78 Ca	260.33 ± 9.89 Cb	620.29 ± 6.50 Aab	592.65 ± 21.50 Aa	419.36 ± 7.52 Ba
	C2	481.43 ± 18.83 Aa	355.73 ± 10.33 BCa	416.80 ± 4.28 ABa	336.72 ± 16.11 Ca	307.08 ± 8.68 Ca	558.44 ± 17.92 Abc	582.20 ± 26.93 Aa	438.77 ± 38.51 Ba
	C3	498.30 ± 18.51 Aa	342.09 ± 2.96 Ca	430.23 ± 10.27 Ba	350.73 ± 15.96 Ca	298.31 ± 15.04 Bab	510.65 ± 20.07 Ac	534.44 ± 29.12 Aa	494.40± 23.25 Aa
non-Karst soil	C0	191.06 ± 10.00 Cc	688.03 ± 17.02 Ca	913.04 ± 38.78 Ba	2492.78 ± 236.33 Aa	109.02 ± 5.14 Ca	259.84 ± 2.82 Bb	96.43 ± 5.02 Ca	322.78 ± 18.78 Aa
	C1	707.18 ± 47.02 Bb	646.73 ± 32.37 Ba	811.68 ± 41.46 Bab	2391.27 ± 135.48 Aab	98.81 ± 2.92 Ca	262.63 ± 5.19 Bb	101.06 ± 2.40 Ca	309.51 ± 10.17 Aa
	C2	710.33 ± 35.1 Bb	666.71 ± 9.31 Ba	768.00 ± 38.44 Bab	2079.16 ± 139.89 Aab	101.98 ± 6.23 Ba	287.86 ± 13.06 Aab	97.54 ± 4.96 Ba	275.74 ± 8.47 Aa
	C3	852.5 ± 25.32 Ba	650.85 ± 22.83 Ba	750.65 ± 35.72 Bb	1798.47 ± 93.23 Ab	101.92 ± 2.46 Ba	294.44 ± 2.53 Aa	103.39 ± 1.45 Ba	286.37 ± 6.30 Aa

Notes: Different capital and lower letters indicate significant differences among different culture days at four biochar treatments.

## Data Availability

The raw data supporting the conclusions of this article will be made available by the authors on request.

## Supplementary Materials

The following supporting information can be downloaded at: https://www.mdpi.com/article/10.3390/microorganisms14061373/s1, Figure S1: Diversity of *phoD* bacteria communities at the order level in the two soil types with biochar amended at application amounts of 0, 5, 10, and 15 t·hm^−2^ (C0, C1, C2, C3, respectively). OTUs in karst soil (a), OTUs in non-karst soil (b), Chaol in karst soil (c), Chaol in non-karst soil (d), Shannon diversity in karst soil (e), Shannon diversity in non-karst soil (f), Simpson diversity in karst soil (g), Simpson diversity in non-karst soil (h). Different capital and lower letters indicate significant differences among different culture days at four biochar treatments; Figure S2: Co-occurrence networks of *phoD*-harboring bacterial community in the two soil types, karst (a–d) and non-karst (e–h), on days 7, 20, 40, and 80 of incubation. Red lines represent positive interactions, and green lines represent negative interactions; Figure S3: Co-occurrence networks of *phoD*-harboring bacteria in the two soil types, karst (a) and non-karst (b), under bagasse biochar addition. Red lines represent positive interactions, and green lines represent negative interactions; Table S1: Parameters of the *phoD* bacteria co-occurrence network analysis for karst and non-karst forest soils under different application amounts of bagasse biochar; Table S2: Parameters of the *phoD* bacteria co-occurrence network analysis for the two soil types under different incubation times; Table S3: Parameters of the *phoD* bacteria co-occurrence network analysis for karst and non-karst forest soils; Table S4: Effects of bagasse biochar addition on phosphorus fractions in karst forest soil and non-karst forest soil.
