# Supplementary material for: Enhancing Phosphorus Availability Through Bagasse Biochar Addition and Changes in phoD Bacterial Communities of Karst and Non-Karst Forest Soils
Source: Microorganisms. 2026 Jun 21;14(6):1373. doi: 10.3390/microorganisms14061373 (PMC13304114; doi:10.3390/microorganisms14061373)
Supplement: Supplementary file 1 [file microorganisms-14-01373-s001.zip › microorganisms-4310857-supplementary.pdf]

Supplementary Materials

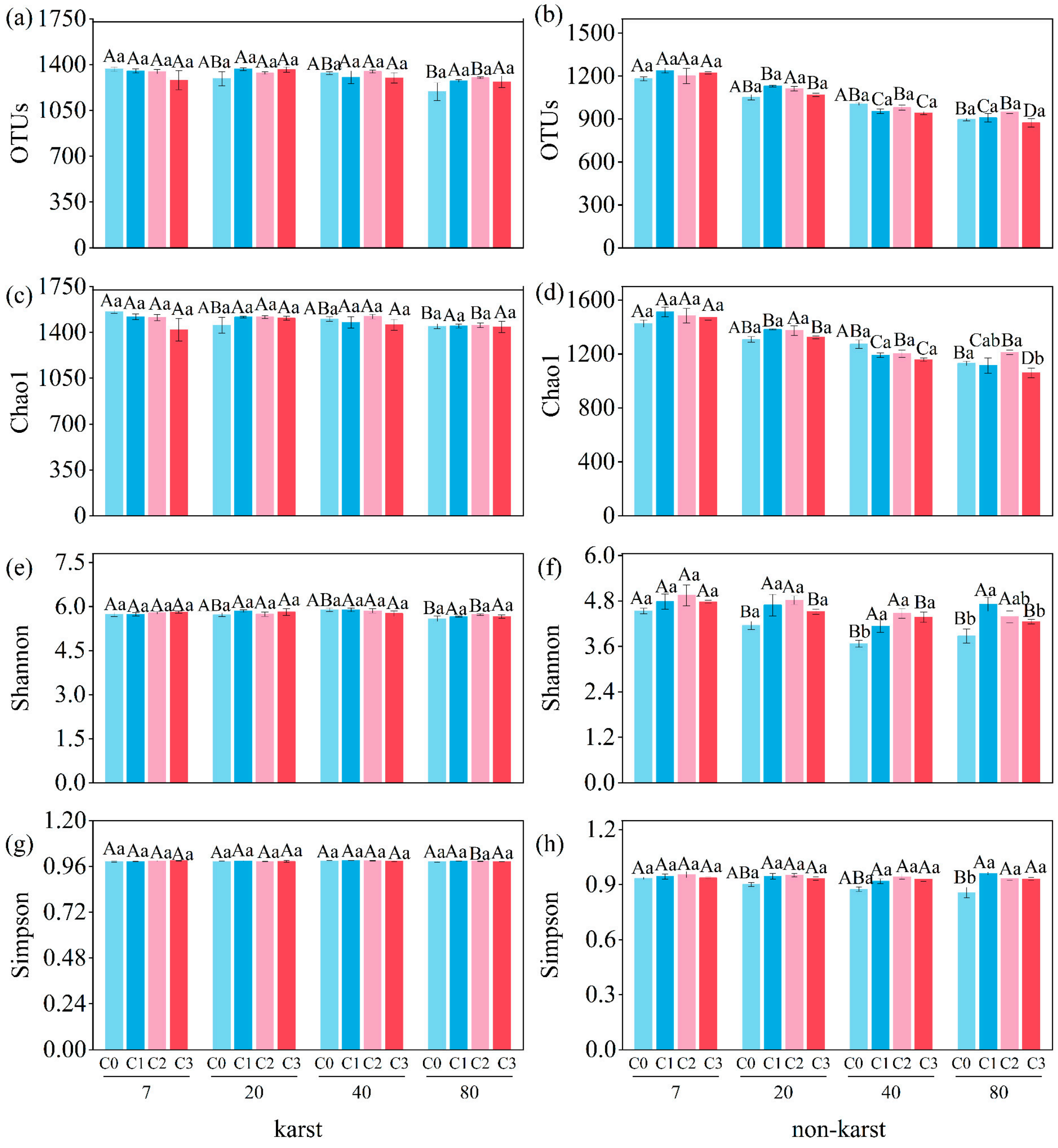

Figure S1. Diversity of phoD bacteria communities at the order level in the two soil types with biochar amended at application amounts of 0, 5, 10, and 15 t·hm-2 (C0, C1, C2, C3, respectively). OTUs in karst soil (a), OTUs in non-karst soil (b), Chaol in karst soil (c), Chaol in non-karst soil (d), Shannon diversity in karst soil (e), Shannon diversity in non-karst soil (f), Simpson diversity in karst soil (g), Simpson diversity in non-karst soil (h). Different capital and lower letters indicate significant differences among different culture days at four biochar treatments.

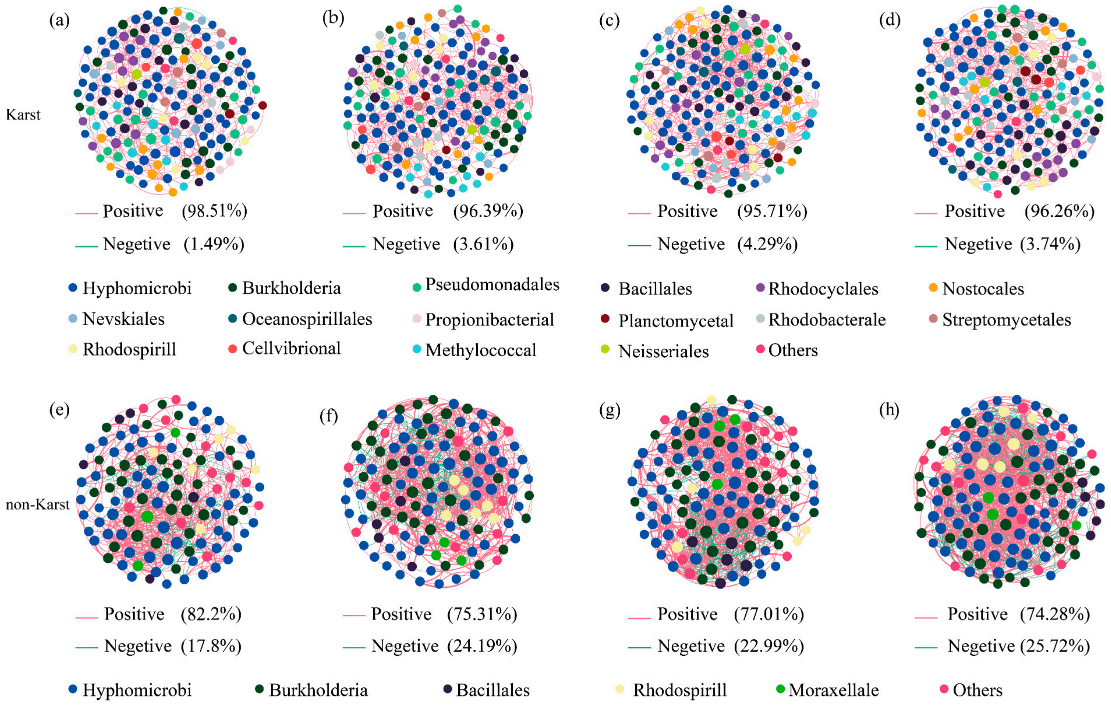

Figure S2. Co-occurrence networks of *phoD*-harboring bacterial community in the two soil types, karst (a, b, c, d) and non-karst (e, f, g, h), on days 7, 20, 40, and 80 of incubation. Red lines represent positive interactions, and green lines represent negative interactions.

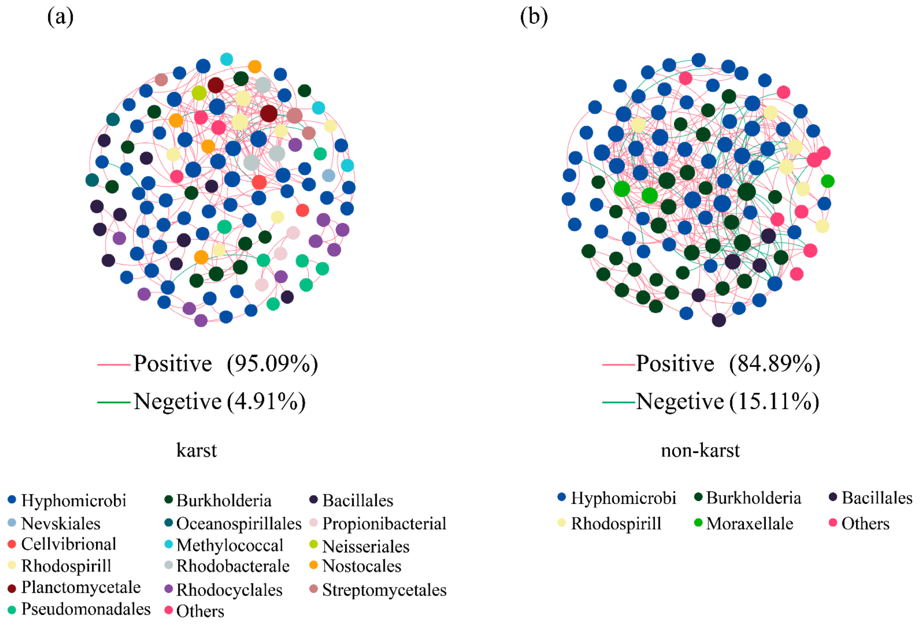

Figure S3. Co-occurrence networks of *phoD*-harboring bacteria in the two soil types, karst (a) and non-karst (b), under bagasse biochar addition. Red lines represent positive interactions, and green lines represent negative interactions.

Table S1. Parameters of the *phoD* bacteria co-occurrence network analysis for karst and non-karst forest soils under different application amounts of bagasse biochar.

Soil type	Bio-char	Notes	Edges	Avg.Degree	Diamter	Density	Clust.Coeff	Avg.Path
karst	C0	175	533	6.091	11	0.035	0.388	4.133
	C1	178	1065	11.966	8	0.068	0.455	3.233
	C2	171	677	7.918	10	0.047	0.475	3.667
	C3	180	774	8.6	9	0.048	0.429	3.493
non-karst	C0	117	365	6.239	11	0.054	0.444	4.062
	C1	124	472	7.613	8	0.062	0.477	3.433
	C2	121	348	5.752	11	0.048	0.458	3.995
	C3	117	336	5.744	10	0.05	0.409	4.087

Table S2. Parameters of the *phoD* bacteria co-occurrence network analysis for karst and non-karst forest soils under different incubation times.

Soil type	Times (day)	Notes	Edges	Avg.Degree	Diamter	Density	Clust.Coeff	Avg.Path
karst	7	173	471	5.445	9	0.032	0.384	4.201
	20	178	581	6.528	10	0.037	0.398	3.777
	40	177	722	8.158	8	0.046	0.392	3.479
	80	174	561	6.448	11	0.037	0.437	4.122
non-karst	7	124	618	9.968	8	0.081	0.455	3.05
	20	125	897	14.352	6	0.116	0.514	2.513
	40	124	957	15.435	6	0.125	0.529	2.545
	80	129	1147	17.783	6	0.139	0.549	2.405

Table S3. Parameters of the *phoD* bacteria co-occurrence network analysis for karst and non-karst forest soils.

Soil type	Notes	Edges	Avg.Degree	Diamter	Density	Clust.Coeff	Avg.Path Length
Karst	120	224	3.733	12	0.031	0.677	4.033
Non-Karst	109	331	6.073	9	0.056	0.544	3.359

Table S4. Effects of bagasse biochar addition on phosphorus fractions in karst forest soil and non-karst forest soil.

Soil type	Treatment	Olsen-P	Citrate-P	HCl-P	Enzyme-P	$CaCl_2$-P	MBP
karst	C1	57.13%	113.15%	284.40%	1.19%	1.00%	23.62%
	C2	169.89%	534.90%	509.95%	4.04%	5.31%	7.00%
	C3	398.81%	754.84%	647.53%	6.74%	6.47%	-30.18%
non-karst	C1	276.70%	258.38%	347.79%	-2.70%	5.33%	89.08%
	C2	711.78%	747.76%	1362.15%	8.19%	9.58%	147.83%
	C3	1052.89%	937.99%	1991.08%	55.41%	40.77%	-25.79%
